# A Novel Behavioral Assay to Investigate Gustatory Responses of Individual, Freely-moving Bumble Bees (*Bombus terrestris*)

**DOI:** 10.3791/54233

**Published:** 2016-07-21

**Authors:** Carolyn Ma, Sébastien Kessler, Alexander Simpson, Geraldine Wright

**Affiliations:** ^1^Institute of Neuroscience, Newcastle University

**Keywords:** Neuroscience, Issue 113, taste, gustation, proboscis extension, insect, bumble bees, *Bombus terrestris*

## Abstract

Generalist pollinators like the buff-tailed bumble bee, *Bombus terrestris*, encounter both nutrients and toxins in the floral nectar they collect from flowering plants. Only a few studies have described the gustatory responses of bees toward toxins in food, and these experiments have mainly used the proboscis extension response on restrained honey bees. Here, a new behavioral assay is presented for measuring the feeding responses of freely-moving, individual worker bumble bees to nutrients and toxins. This assay measures the amount of solution ingested by each bumble bee and identifies how tastants in food influence the microstructure of the feeding behavior.

The solutions are presented in a microcapillary tube to individual bumble bees that have been previously starved for 2-4 hr. The behavior is captured on digital video. The fine structure of the feeding behavior is analyzed by continuously scoring the position of the proboscis (mouthparts) from video recordings using event logging software. The position of the proboscis is defined by three different behavioral categories: (1) proboscis is extended and in contact with the solution, (2) proboscis is extended but not in contact with the solution and (3) proboscis is stowed under the head. Furthermore the speed of the proboscis retracting away from the solution is also estimated.

In the present assay the volume of solution consumed, the number of feeding bouts, the duration of the feeding bouts and the speed of the proboscis retraction after the first contact is used to evaluate the phagostimulatory or the deterrent activity of the compounds tested.

This new taste assay will allow researchers to measure how compounds found in nectar influence the feeding behavior of bees and will also be useful to pollination biologists, toxicologists and neuroethologists studying the bumble bee's taste system.

**Figure:**
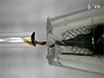


## Introduction

Plant-pollinator interactions are complex. Pollinators visit flowers to obtain nectar and pollen as food; in turn, pollinators facilitate sexual reproduction in plants. While this relationship is mostly mutualistic, floral nectar and pollen sometimes contain toxins or other plant compounds^1-5^****which can harm pollinators. The ecological rationale for the presence of such compounds in nectar and pollen is not clear in all settings. One outstanding question in this field is how pollinators such as bees can detect and avoid flowers with nectar containing toxins.

The bumble bee species, *Bombus terrestris *(Linnaeus, 1758), is a generalist pollinator that visits the flowers of many plant species including those producing nectar containing toxins^6^. Bumble bees have been shown to avoid consuming solutions containing high concentrations of toxins in a 24 hr two-choice assay^7^. This assay of food consumption described by Tiedeken *et al*.^7^ revealed that bees can detect bitter compounds in solutions. However, this assay was unable to distinguish taste from post-ingestive processes such as malaise that could also affect feeding behavior over this time interval^8-10^.

Bees possess gustatory sensilla on their antennae, mouthparts and tarsi to detect compounds^11-13^. The proboscis extension reflex (PER) experiments involve restraining individual bees in a harness and then stimulating the bee's antennal sensilla to produce the feeding reflex^14-17^. Bees can be restrained in individual harnesses and then stimulated to produce the feeding reflex as an assay of their ability to taste compounds^18,19^. Others have modified the PER assay to study the sensitivity of the antennae or mouthparts to toxins^9,20^. However, bees are subjected to stress during harnessing. This could affect how they respond to compounds^21^.

Here, a new assay is described to assess the behavioral taste response of freely-moving bumble bees to sucrose and quinine, an alkaloid that has previously been reported to be deterrent^9^ and toxic^10^ to honey bees (*Apis mellifera*) and bumble bees (*Bombus terrestris*)^7,^^22^. Although quinine has not been found in plant nectar, this alkaloid is often used as an aversive stimulus in behavioral and physiological studies in bees^7,9,12,13,22^. The method involves video recording the bumble bees' mouthparts at great resolution during the initial proboscis contact with the test solutions. Specifically, the fine structure of the feeding response is examined by continuously scoring behavior over a 2 min interval. The volume of solution consumed is measured during the feeding period and so the amount of food eaten can be correlated with the microstructure of the feeding behavior. Also the speed of the proboscis retraction is measured, as an indicator of an active avoidance, and therefore pre-ingestive detection.

## Protocol

### 1. Capturing Bees from the Colony and the Starvation Period

Note: Experiments described here were performed at Newcastle University, UK with *Bombus terrestris audax*. Multiple (2-3) commercially purchased colonies were used per treatment. The colonies have been maintained on a bench at laboratory conditions (25 ± 2 °C and 28 ± 2% RH) in constant darkness and were fed with honey bee collected pollen and sugar solutions *ad libitum*.

1. Collect individual worker bumble bees using a plastic vial (7 cm long, 2.8 cm inner diameter) with a perforated plastic stopper, after having opened the gate to the colony just long enough for one bee to exit and be trapped.
2. Prior to the experiment, individually starve all bumble bees for 2-4 hr in the plastic vials and keep at room temperature in complete darkness.

### 2. Transferring Bees into the Holding Tubes and the Habituation Phase

1. After the starvation period, transfer a bumble bee directly from the plastic vial into a holding tube. The holding tube is a modified 15 ml centrifuge tube (length: 119 mm; diameter 17 mm), with a 4 mm hole drilled at the tip and a piece of steel mesh (base: 8 mm; height 30 mm) fixed inside by melting the plastic of the tube with a heated dissecting steel needle.
2. Fix the holding tube containing the bumble bee onto a polystyrene holder with dental wax. Fix two pieces of cardboard on either side of the holding tube. This is to shield the bee from visual stimuli that might interfere with the experiment.
3. Position a digital microscopic camera 5 cm above the tip of the holding tube and connect the camera to a compatible laptop.
4. Adjust the holding tube so that at least the first 18 mm of the holding tube tip is within the video frame. Prior to the experiment, commence the 3 min habituation period.

### 3. Pre-test Phase: Presenting a Drop of Sucrose

1. Connect a syringe to a female adapter containing a droplet of sucrose solution (~3.5 µl, 500 mM sucrose dissolved in deionized water). Present the sucrose inside the holding tube tip to motivate the extension of the proboscis.
2. Give the bumble bee up to 5 min to consume the sucrose droplet. If the droplet is not consumed, remove the bumble bee from the experiment.
3. Begin video recording after the habituation period. In this study, the proboscis activity was recorded at 26.7 frames/sec^-1^ with a 25X magnification rate.

### 4. Test Phase: Presenting the Test Solution

1. Fill a 100 µl microcapillary tube with the test solution. Connect it to a piece of silicone tubing (6 cm length, 1 mm inside diameter) and fix it to a micro manipulator.
  1. Connect the tubing via a male adapter to another silicone tubing (6 cm length, 4 mm inside diameter), which acts as a pipette bulb.
  2. Position the microcapillary tube 5-10 mm away from the holding tube tip. Gently squeeze the tubing to maintain the feeding solution at the tip of the microcapillary tube.
2. After the bumble bee consumes the sucrose droplet, immediately remove the syringe containing the 500 mM sucrose solution.
3. Begin the 2 min test phase when the bumble bee's proboscis contacts the solution inside the microcapillary tube.
  1. To control for possible evaporation, fill two additional microcapillary tubes with sucrose or water and manipulate it exactly as during the test phase.
4. Before and after each trial scan the liquid levels inside the microcapillary tube using a scanner at 600 dpi to measure the amount of food consumed (**Figure 4A**).

### 5. Image Analyses

1. Determine the volume of solution consumption using ImageJ (version 1.48), an image processing software.
  1. Upload the image file and zoom into the image (~400%). To set the reference scale, select the straight line tool and draw a line between the two ends of the microcapillary tube. Select 'Analyze' then 'Set Scale'. Input the total length of the tube under 'Known distance' and the corresponding unit under 'Unit of length'.
  2. Select the straight line tool again and draw a line between the two ends of the liquid level. Select 'Analyze' then 'Measure'. In the results window the length of the liquid is given under the 'Length' column.
2. Calculate the volume of solution consumption by using the formula: 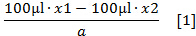 where 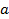 is the length of the microcapillary tube and 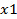 and 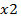 are the measured lengths of the liquid inside the microcapillary tube before and after the test phase, respectively.

### 6. Video Analyses

1. Score the feeding behaviors during the 2 min testing phase of each video using an event logging software (See **Materials Table**).
  1. At first, define the feeding behaviors (*i.e*. the elements) in the behavioral classes menu of the recording software. The feeding behaviors are as followed:****(1) proboscis out/contact: the proboscis extends and is in contact with the solution inside the microcapillary tube (2) proboscis out/no contact: the proboscis extends and is not in contact with the solution inside the microcapillary tube, (3) proboscis stowed: the proboscis is not extended but instead stowed under the head and (4) out of sight: the bumble bee is out of the video frame.
  2. Set each behavior as a 'state' and 'mutually exclusive' in the properties menu and make continuous recordings for a 2 min interval. Replay the videos in slow-motion mode (2 times slower) for more precision.
2. Measure the speed of proboscis retraction from the test solution after the first contact between two consecutive frames (separated by 37.5 msec in the video recordings shown here) using a motion tracking video software (See **Materials Table**).
  1. Upload the video file and skip to the frame where the proboscis first contacts the solution.
  2. To set the reference scale, select the line tool and draw a line on the width of the microcapillary tube in the video frame. Right click on the line and select 'Calibrate measure'. Input the width of the capillary tube and the corresponding unit.
  3. Select 'Image' then 'Coordinate system origin'. On the new window click on the tip of the proboscis and select 'Apply'.
  4. Select the hand move tool, right click on the tip of the proboscis on the video frame and select 'Track Path'. Move to the next frame and readjust the tracking point to the tip of the proboscis.
3. Right click on the tracking point and select 'Configuration'. Select 'Complete Path' and select 'Speed' under measurement. Select 'Apply'. The speed is then displayed.

## Representative Results

The novel assay is used to test the feeding responses to 1 M sucrose, 1 M sucrose solution plus 1 mM quinine and deionized water alone. The immediate feeding responses to each treatment are determined by quantifying the duration of proboscis contacts with the test solution, the frequency of the feeding bouts and the speed of the proboscis retracting away from the test solution after the first contact during the 2 min test phase. The volume of solution consumed is also measured after the test phase. In this study, we have chosen a bout criterion interval of 5 sec (**Figure 1, **see **Supplemental File**) based on previous work by French *et al*.^25^ who used a 5 sec threshold to characterize the proboscis retraction behavior by *Drosophila* in response to deterrent compounds^25^. Thus, we defined a feeding bout as a contact between the extended proboscis and the solution not interrupted by an absence of contacts of 5 sec or more.

In comparison to sucrose and deionized water alone, adding quinine to sucrose solution evidently deters feeding by bumble bees as they will rapidly move away if they detect an aversive substance (**Video Figure 1**).

In this experiment, the treatments have a significant effect on the cumulative duration of proboscis contacts during the test phase (ANOVA on the log-transformed data, F_2,31 _= 41, *p *<0.001). The cumulative duration of contact time with sucrose containing quinine is significantly reduced in comparison to sucrose alone (*p *<0.001) but not to deionized water alone (*p *= 0.219) (**Figure 2**). Similarly, the treatments have a significant effect on the cumulative duration of feeding bouts (ANOVA on the log-transformed data, F_2,31 _= 27.95, *p* <0.001, **Figure 3A**). The cumulative duration of feeding bouts with sucrose containing quinine is significantly reduced in comparison to sucrose alone (p <0.001) but not compared to deionized water alone (p = 0.41). The treatments have also a significant effect on the frequency of feeding bouts (Poisson GLM with a log link function, change in deviance compared to the *c*^2^ distribution: *p *<0.050), whereby the number of bouts with sucrose containing quinine is significantly higher in comparison to sucrose (*p *<0.01) but marginally significantly different to the deionized water treatment (*p *= 0.055, due to one bumblebee displaying seven feeding bouts on water, **Figure 3B**). Likewise, the speed of proboscis retraction differs significantly between treatments (ANOVA on the log-transformed data, F_2,31 _= 5.12, *p *<0.050). Bumble bees retract the proboscis away from the test solution significantly faster after the first contact with sucrose containing quinine than with sucrose or deionized water alone (*p *<0.050, **Figure 3C**). These results suggest that quinine triggers an active avoidance behavior in bumble bees. The treatments also have a significant effect on the total volume of solution consumed (ANOVA on the log-transformed data, F_2,32 _= 62.5, p <0.001), whereby the consumption of sucrose containing quinine is reduced in comparison to sucrose (*p* <0.001) but not to deionized water (*p* = 0.457) (**Figure 4B**). The volume of solution evaporated from the capillary during the test period is negligible. At laboratory conditions (25 ±2°C and 28 ±2% RH), the evaporation varies between 0.033 to 0.883 µl with an average of 0.276 µl and 0.171 µl for deionized water and 1 M sucrose respectively.

In this assay contacts between the antenna and the test solution cannot be prevented. Nonetheless, the percentage of bumble bees using their antennae to taste the feeding solution during the test phase (sucrose: 46.1%, sucrose plus quinine: 60.0% and deionized water: 33.3%) is not significantly different between the treatments (binomial GLM, change in deviance compared to the *c*^2^ distribution: *p *= 0.450*)*. No effect of the treatments is found on the latency between the first antennal contacts and the test solution and the first contacts of the proboscis (median: 2.67 sec for sucrose; 1.10 sec for sucrose plus quinine; 0.80 sec for deionized water, ANOVA on the log-transformed data, F_2,13 _= 0.620, *p *= 0.550). In addition, the percentage of bumble bees extending the proboscis to taste the test solution remains constant across the treatments (sucrose: 66.7%; sucrose plus quinine: 50.0%; deionized water: 52.2%; binomial GLM, change in deviance compared to the *c*^2^ distribution: *p *= 0.840). Together these results suggest that the antennae play a minor role in the detection of the toxins in this assay.

A separate experiment examines whether it is necessary to test bees for a period of time longer than 2 min. The amount of food consumed by bees is tested with the 1 M sucrose or 1 mM quinine in 1 M sucrose solutions in two conditions: a 2 min test period and a 10 min test period. For both treatments, total food consumption does not differ for the test periods and no significant interactions occur between the test period and the treatment (N = 6 - 13, ANOVA on the log transformed data; effect of the treatments:* F*_1,31 _= 54.8, *p *<0.001; effect of the test period: F_1,31_= 0, *p *= 0.979; effect of the interaction: F = 0.1, *p *= 0.457). In summary, a 2 min test period is sufficient to assess the effect of the solution on the total amount of food consumed by bumblebees and the deterrent effects of toxic or repellent substances in this assay. Thus, by measuring food consumption and assaying feeding behavior, it is possible to correlate total food consumption to the fine structure of feeding during the assay.


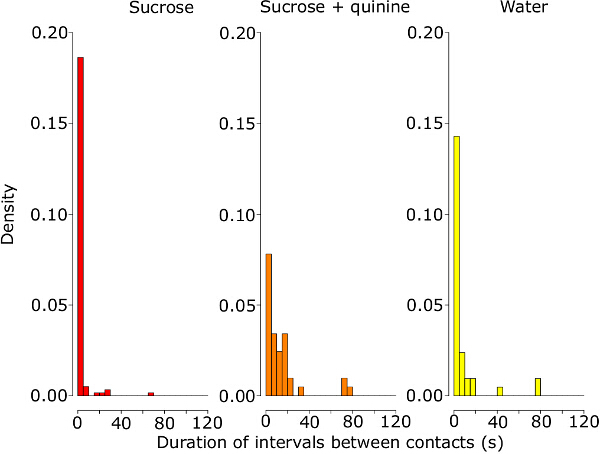
**Figure 1: Latency Periods between the Proboscis Contacts during the First 2 Min of the Feeding Assay.** Density plots of the time latency periods separating each proboscis contact with the 1 M sucrose solution, the 1 M sucrose + 1 mM quinine solution and water. The cumulative data from 13, 10 and 11 bees are represented respectively. Please click here to view a larger version of this figure.


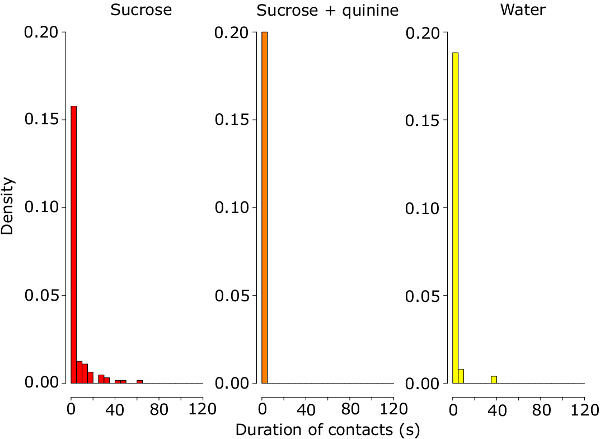
**Figure 2:Proboscis Contact Durations during the First 2 Min of the Feeding Assay.** Density plots of the proboscis contact durations by bumblebees feeding on 1 M sucrose, 1 M sucrose + 1 mM quinine or water. Sample size as in Figure 1. Please click here to view a larger version of this figure.


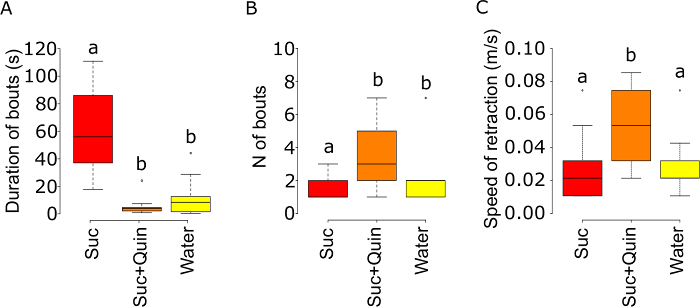
**Figure 3: Proboscis Activity of Bumblebees Feeding on 1 M Sucrose, 1 M Sucrose + 1 mM Quinine or Water. **(**A**) The cumulative duration of feeding bouts during the test phase (**B**) the frequency of feeding bouts and (**C**) the speed of the proboscis retraction after first contact. Lettering indicates a significant difference: treatments with different letters indicate P <0.05. Box plots represent the median (black bars), the lowest and the highest data points still within 1.5 of the interquartile range (whiskers) and the outliers (circles). Sample size as in Figure 1. Please click here to view a larger version of this figure.


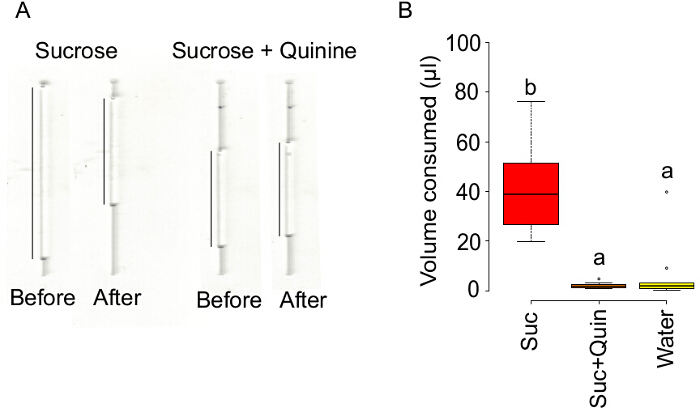
**Figure 4: Quinine supresses feeding by bumble bees. **(**A**) Scanned images of the microcapillary tubes showing the level of the 1 M sucrose or the 1 M sucrose plus 1 mM quinine solution (indicated by a black line) before and after the test phase respectively. (**B**) Consumption of 1 M sucrose, 1 M sucrose plus 1 mM quinine or deionized water alone by bumble bees after the test phase. Lettering indicates a significant difference: treatments with different letters indicate P <0.001. Box plots represent the median (black bars), the lowest and the highest data points still within 1.5 of the interquartile range (whiskers) and the outliers (circles). Sample size as in Figure 1. Please click here to view a larger version of this figure.

**Video Figure 1: **Video Recordings of the Proboscis Activity toward (**A**) 1 M Sucrose, (**B**) 1 M Sucrose Plus 1 mM Quinine and (**C**) Deionized Water during the Test Phase.

## Discussion

With this novel behavioral assay, quinine is shown to deter feeding of the buff-tailed bumble bee. The reduced proboscis contact time and feeding bout frequency with water or the sucrose solution laced with quinine is interpreted here as a refusal to initiate further feeding on non-nutritive or potentially toxic solutions. When quinine is added to 1 M sucrose solution, bumble bees not only reduce the volume of solution they consume, they also retract the proboscis faster, thus reducing contact time between the mouthparts and the solution containing a toxin. Together, these results suggest that quinine is perceived by the gustatory receptor cells on the mouthparts of the bumble bee, as already previously identified in the honey bee^9^. Quinine is a toxin for insects that induces malaise-like behavior in the honey bee^10^ and knockdown in the malaria mosquito (*Anopheles gambiae*)^23^. This assay could well lead to the identification of some deterrent and potentially toxic compounds that are perceived by the taste receptor cells on the mouthparts in the bumble bees.

It is crucial for the microcapillary tube to be filled with a sufficient volume of test solution to last throughout the test phase. It is recommended that at least around three quarters of the microcapillary tube (*e.g.* 70-80 µl) is filled. However, care should be taken to not completely fill the microcapillary tube to reduce the risk of spillage during the process of scanning and attaching the microcapillary tube to the experimental apparatus. Care should also be taken when presenting the 500 mM sucrose droplet to the bumble bee, so that the experimenter avoids leaking the droplet into the holding tube.

The 4 mm hole at the tip of the holding tube is large enough for an adult worker bumble bee to naturally extend its proboscis toward the test solution. However it is possible that bumble bees can taste the solution with their antennae before extending their proboscises. This might affect the probability of proboscis extension as PER could be elicited in bumble bees by stimulating their antennae with a sugar solution^15^. In fact the antennae of Hymenoptera like the parasitoid wasp (*Trissolcus brochymenae*)^24^ or the honey bee^13^ are equipped with taste sensilla, allowing them to taste sugars and toxins like quinine. Consequently, initial antennal contacts with solutions containing highly deterrent compounds like quinine could also reduce the motivation of a bumble bee to extend its proboscis and therefore affect the experimental success rate. Although antennal contact with the test solution cannot be controlled, in the present study we did not find any significant effect of antennal contact on proboscis extension toward the test solution. In this assay, immediately setting up the microcapillary tube after the pre-test phase when the bumble bees' antennae are still within the holding tube can reduce the opportunity for the bumble bees to taste the test solution with their antennae.

The main limitation of this assay arises when tracking the proboscis retraction away from the test solution after the first proboscis contact using the motion tracking video software. The video footage only displays 2D movement of the proboscis, so the given output of the speed measurement can be under or over estimated. However with some modifications, this aspect of the assay could be improved.

This assay can be used to observe natural feeding responses toward solutions containing different compounds including natural-occurring plant secondary metabolites. Observing the immediate feeding responses with this assay gives detailed information about how bumble bees detect these compounds. This is advantageous over existing 'go-no go' methods like PER^18,19^ and over two-choice assays^7^ because this method produces several behavioral response measures including food consumption during a discrete feeding bout.

Measuring several parameters simultaneously allows a better evaluation of the palatability of a compound. For example in our assay, bumble bees avoid consuming water or the sucrose solution laced with quinine. Retraction of the proboscis could be caused by a change in the responses of the sugar receptor cells^12,13^. Our assay shows that bumble bees retract the proboscis faster after contacting the sucrose plus quinine solution than water alone; this could suggest that quinine affects a distinct set of neurons in addition to inhibiting sugar sensing neurons^9,12,13,25^.

Our assay permits the analysis of the temporal pattern of behavioral responses during feeding. A similar protocol where the consumption time and the number of bouts is measured has already been implemented to evaluate the feeding response of *Drosophila* to nutritive and non-nutritive sugars^26^. We predict that bees will exhibit a more reliable response to feeding stimulants in our assay than in other methods such as PER because the bees are free to move in the holding tube^21^. This technique will permit an exhaustive analysis of the taste thresholds for nutrients and toxins to illuminate the mechanisms of feeding in bumble bees and potentially other bee species.

## Disclosures

The authors declare no conflict of interest.
